# GRAY FOR A DAY: A SIMULATION ACTIVITY IMPACTS PARTICIPANTS IN COMMUNITY-BASED HEALTH EDUCATION

**DOI:** 10.1093/geroni/igad104.2705

**Published:** 2023-12-21

**Authors:** David Buys, Jasmine Harris-Speight, Pamela Redwine, Ann Sansing, Erin Yelland

**Affiliations:** Mississippi State University, Mississippi State, Mississippi, United States; Mississippi State University, Starkville, Mississippi, United States; Mississippi State University, Starkville, Mississippi, United States; Mississippi State University, Starkville, Mississippi, United States; Kansas State University, Manhattan, Kansas, United States

## Abstract

Nearly 80% of older adults own their own homes; approximately two-thirds live within 25 miles of relatives; and most prefer to age-in-place, without access to geriatric-specific care. They rely on primary care clinics and family, friends, and other local support to meet their health needs. The healthcare workforce and lay public need to better understand age-related functional decline. Simulation activities can increase one’s understanding of new concepts or perspectives. One such activity, “Gray for a Day,” offers a didactic presentation and simulations of functional decline. In 2022, 10 Mississippi State University Cooperative Extension agents offered the “Gray for a Day” training to 550 individuals across 35 sessions in community colleges, high school career and technical education classes, and hospitals and nursing home food service departments. Participant evaluations were collected to assess learning outcomes and empathy development. Process evaluation data and descriptive statistics are reported. Nearly all (98%) of the participants reported increases in knowledge of age-related sensory and functional decline and increases in understanding how age-related sensory and functional decline can impact one’s daily life; and 95% reported an increase in considering how they can better interact with older adults experiencing functional decline. Understanding challenges facing older adults is essential to empathy and important both in a home environment and in the workplace. The “Gray for a Day” simulation activity is feasible for implementation in multiple settings and has the potential to increase knowledge, understanding, and possibly empathy for older adults experiencing functional decline.

